# Clinical Outcomes and Safety Profile of Vancomycin in Outpatient Parenteral Antimicrobial Therapy Services: A Systematic Review

**DOI:** 10.3390/antibiotics15060630

**Published:** 2026-06-22

**Authors:** Moska Hassanzai, Ramon R. Contrucci, Birgit C. P. Koch, Nelianne J. Verkaik, Brenda C. M. de Winter, Hein A. W. van Onzenoort

**Affiliations:** 1 Department of Hospital Pharmacy, Erasmus MC University Medical Center, 3015 GD Rotterdam, The Netherlands; 2 Department of Clinical Pharmacy, Amphia Hospital, 4818 CK Breda, The Netherlands; 3 Department of Clinical Pharmacy, Dijklander Hospital, 1624 HP Hoorn, The Netherlands; 4 CATOR: Center for Antimicrobial Treatment Optimization, 3015 GD Rotterdam, The Netherlands; 5 Department of Clinical Pharmacy and Pharmacology, University Medical Center Groningen, University of Groningen, 9713 GZ Groningen, The Netherlands; 6 Department of Medical Microbiology and Infectious Diseases, Erasmus MC University Medical Center, 3015 GD Rotterdam, The Netherlands; 7 Department of Pharmacy, Pharmacology and Toxicology, Radboud University Medical Center, Radboud Institute for Health Sciences, 6525 GA Nijmegen, The Netherlands

**Keywords:** vancomycin, OPAT, infectious diseases, systematic review, effectiveness of antibiotic therapy, safety to antibiotic therapy

## Abstract

**Introduction:** Vancomycin is a widely used antibiotic in Outpatient Parenteral Antimicrobial Therapy (OPAT) services. The objective of this systematic review was to evaluate the published literature on the efficacy and safety outcomes of outpatient vancomycin therapy. **Methods:** A systematic search was performed in Embase, Medline ALL, the Web of Science Core Collection, and the Cochrane Central Register of Controlled Trials from database inception until 20 March 2026. Both randomized controlled trials and non-randomized studies published in peer-reviewed journals were included. Study quality was assessed using the Newcastle–Ottawa Scale. **Results:** A total of 75 studies were included. Clinical success rates of 40.9% to 100% were reported. Reported adverse event (AE) rates ranged widely from 5.7% to 85.7%. Comparative studies suggest a higher risk of nephrotoxicity during intermittent infusion compared to continuous infusion. Reported line-related AE ranged from 1.1% to 5.7% and readmission risks associated with vancomycin use were inconsistent across studies. **Conclusions:** This systematic review shows that vancomycin is an effective agent to use in OPAT setting, however its use is associated with a risk of adverse events. The findings of this study underscore the need for a dedicated multidisciplinary OPAT team to ensure proper follow-up and tailored vancomycin management in the outpatient setting.

## 1. Introduction

Outpatient Parenteral Antimicrobial Therapy (OPAT) was first introduced in 1974 in the United States and has been defined by the Infectious Diseases Society of America (IDSA) as ‘the provision of parenteral antimicrobial therapy in at least two doses on different days without intervening hospitalization’ [1,2]. OPAT services have become increasingly common worldwide with a significant growth over the past couple of decades [3,4,5,6,7,8,9]. OPAT offers the opportunity for early hospital discharge and is associated with greater comfort for the patient, higher patient satisfaction, lower risk of nosocomial complications and an important cost reduction for the health care system [10,11,12,13,14,15].

A widely used drug in OPAT is vancomycin (VAN). VAN is used in the treatment of bacterial infections caused by gram-positive bacteria (e.g., methicillin-resistant *Staphylococcus aureus*, coagulase-negative staphylococci, and *Enterococcus* species) with an average treatment duration of 6 to 12 weeks, which can often be completed outside the hospital [16,17]. The use of VAN in OPAT services comes with several advantages, such as its low costs, dosing characteristics, and long pharmaceutical stability [3,5,18]. Nevertheless, the use of VAN in OPAT services also poses some challenges. First, VAN has a narrow therapeutic index and is associated with potential adverse events such as hypotension, tachycardia, phlebitis, nephrotoxicity, and hematological toxicity [19,20]. Moreover, the practice of OPAT may increase the risk of adverse events since patients are treated outside the hospital due to limited medical personnel support and less comprehensive clinical monitoring, often conducted remotely [21]. Second, to ensure efficacy and avoid toxicity, therapeutic drug monitoring (TDM) is needed for treatment with VAN [22,23]. Performing TDM in OPAT settings can be challenging due to factors such as patient immobility, nonadherence to treatment plans, geographic isolation, limited transmural communication and restricted access to laboratory facilities [16]. Previous studies have demonstrated that patients discharged with medication subjected to TDM, have an increased risk for readmission during OPAT [24,25,26].

At present, a comprehensive understanding of the risks and benefits associated with VAN use in the outpatient setting is incomplete. We therefore performed a systematic review to identify, systematically evaluate, and summarize the available evidence pertaining to the effectiveness and safety of VAN in the outpatient setting.

## 2. Results

### 2.1. Study Selection

**Figure 1:**
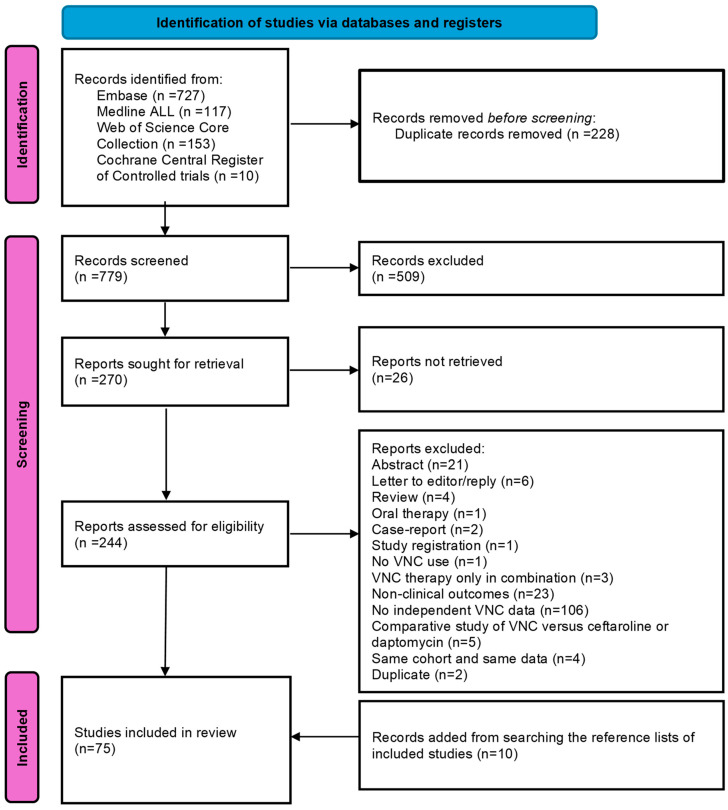
In- and exclusion of papers in systematic review.

A total of 1007 citations were identified with 779 citations remaining after duplicates were removed (Supplementary File S2). A total of 509 studies were excluded by screening titles and abstracts, resulting in a full-text review of 270 studies. After applying inclusion and exclusion criteria we included 65 studies. A manual search of reference lists of included studies and review articles yielded an additional 10 publications. A final total of 75 studies were included in the systematic review. Figure 1 shows the inclusion–exclusion process according to the Preferred Reporting Items for Systematic reviews and Meta-Analysis (PRISMA) flow diagram [27]. The key characteristics and the main results of the included studies are summarized in Table 1 for OPAT studies in VAN patients (18 studies) and in Table 2 for general OPAT studies that report VAN outcomes (57 studies).

### 2.2. Study Characteristics

Of the 75 studies included in this review, 18 studies examined VAN as the sole primary treatment agent in an outpatient setting (Table 1). The other 57 studies reported outcomes in overall OPAT populations, with no focus on a particular antimicrobial agent (Table 2).

The majority of the OPAT studies performed in VAN patients were retrospective studies (16/18, 89%) with the remaining two studies (11%) prospective (Table 1). Five studies (5/18, 28%) compared I-I with C-I of VAN [28,35,41,43,44]. All studies were performed in adult patients with varying indications, but orthopedic infections were the most frequently described. TDM of VAN was performed in all studies, with once-weekly being the most common frequency.

The general OPAT studies were mainly (if specified) retrospective studies (42/55, 76%). Seven studies were performed in specific OPAT populations (one study in IV drug users, five studies in the pediatric population, and one study in hemodialysis patients [50,52,60,61,76,82,92]. The treating indications during OPAT varied extensively across those studies, with 10 studies (18.2%) focusing on specific indications [57,62,63,66,68,78,84,85,89,90]. Variable OPAT models were described in the studies with most combining different delivery models, but 10 (18.2%) studied exclusively HITH [49,54,57,58,66,76,77,84,92,97]. Monitoring (laboratory and TDM monitoring), varied considerably across the included studies with several studies not mentioning any monitoring conditions, and a portion (eight studies, 14.5%) specifying that TDM of VAN was performed [45,53,60,61,64,72,74,93].

### 2.3. Quality Assessment

Overall, the average NOS score of included studies was 6.9 out of 9 (range 6 to 8) for eight cohort studies, 4.8 out of 6 (range 3–6) for nine non-comparative cohort studies and 9 out of 9 for one case-control study (Supplementary File S3). Twelve studies were rated as having a low risk of bias [16,29,30,32,33,34,36,37,38,39,41,42] while the other studies were rated as moderate risk of bias [28,31,35,40,43,44]. The moderate risk of bias was, amongst others, due to confounding.

### 2.4. Effectiveness of Vancomycin in Outpatient Setting

Two studies performed in VAN patients reported clinical success rates of 94.7–100% (Table 1), while a third study in VAN patients found an overall lower cure rate (65.7%) in VAN hemodialysis patients [16,30,32]. Three studies compared the outcomes of continuous infusion (C-I) of VAN versus intermittent infusion (I-I) of VAN in the OPAT setting [41,43,44] (Table 1). These studies found no statistically significant difference in rate of clinical cure rates. In addition, Rees et al. found no significant difference in clinical success between area under the curve (AUC)-based goal trough dosing versus traditional trough dosing [40].

Additionally, two general OPAT studies reported on VAN effectiveness outcomes, with treatment success rates of 40.9% and 94% [52,93] (Table 2).

### 2.5. Safety Profile of Vancomycin in Outpatient Setting

Several studies reported general incidences and risks of adverse events of VAN use in the outpatient setting (Table 1 and Table 2). VAN-related adverse events (AE) rates were reported of 5.7–85.7% [16,52,54,59,60,61,71,74,82,84,91,92,93,96]. Five studies reported an increased risk of VAN use for developing AE’s, risks ranged from 1.7–2.19 (expressed as aIRR, aRR, aOR, OR) [25,51,71,94,95]. Contradictingly, Pulcini et al. found a non-significant odds ratio (OR) of 1.17 (*p* = 0.71) [84].

#### 2.5.1. Nephrotoxicity

One major adverse event associated with VAN use is nephrotoxicity, which typically manifests as acute kidney injury. Different studies and meta-analyses suggest that continuous infusion of vancomycin, avoiding the peak concentration, may be associated with a lower incidence of nephrotoxicity compared to intermittent dosing [98,99]. Four studies compared nephrotoxicity rates in C-I versus I-I VAN outpatients [28,35,41,44]. Shakenerah et al. found that the risk of nephrotoxicity during I-I was 3.22-fold higher (*p* = 0.027), Ingram et al. found statistically higher nephrotoxicity rate during I-I (11.6% versus 23.6%, *p* = 0.067). Vuagnet et al. found that creatinine increased by a mean of 0.4 umol/L/day in I-I group (*p* = 0.02). Contradictory, Benefield et al. found no difference in proportion of patients experiencing nephrotoxicity between C-I and I-I I (9.3% and 7.2% resp., aHR 0.72 (95% CI 0.35–1.50)). Other, non-comparative, VAN studies reported nephrotoxicity outcomes during C-I infusion (four studies) and during I-I infusion (two studies) and found nephrotoxicity rates of 3.6–33% and 7–28%, respectively (Table 1) [29,34,36,37,38,42]. Furthermore, two studies in VAN outpatients compared AUC-based versus trough dosing [31,40]. Both studies found that nephrotoxicity was significantly lower in the AUC cohort.

#### 2.5.2. Line-Related Adverse Events

Due to the relatively low pH of vancomycin, local phlebitis is a VAN-specific adverse event. To minimize vein irritation VAN is often administrated through a central line, allowing for safer administration. The risk of line-related adverse events, including mechanical or infectious complications, is higher in the OPAT setting due to the longer treatment duration, less frequent clinical monitoring, and variable technique of administration [87,100].

In a prospective cohort study with C-I VAN, with the vast majority having a peripherally inserted central catheter (PICC) line (94.3%), a low catheter-related AE rate (5.7%) was reported in 34 VAN OPAT episodes (Table 1) [16]. A retrospective cohort study found, with almost half of the VAN courses administered as combination therapy, three of two hundred and seventy-five VAN courses (1.1%) IV-line associated bacteremia leading to readmission [32].

Another six studies did mention specific information about line-related events for different antibiotics in OPAT setting, including VAN [25,47,61,68,70,87]. Three studies reported a risk ratio and an increased risk with VAN use [25,47,70]. One study (PICC in 71.1%) reported that VAN was associated with catheter complications (aIRR: 2.32, 95% CI: 1.20–4.46) while the other two studies reported a non-significant RR of 1.5 (95% CI 0.9–2.4) for VAN treatment for vascular access failures (PICC in 52%) and a non-significant OR of 3.0 for other line-events (95% CI 0.5–16.6) respectively (use of midlines, PICCs, and tunneled central venous catheters) [25,47,70].

#### 2.5.3. Other Adverse Events

Pai et al. investigated 14 VAN-induced neutropenia cases with 100 controls. VAN was in all these cases discontinued, but the patients did not require hospitalization. Through levels were not associated with development of neutropenia (Table 1) [39]. Two general OPAT studies reported a 5% and 3.9% incidence of neutropenia in VAN-treated outpatients (Table 2) [65,75], whilst one general OPAT study reported an OR of 1.10 (CI 0.75–1.61, *p* = 0.62) for developing neutropenia [46].

Three studies primarily investigated the rate of allergic reactions during general OPAT therapies [48,56,73]. Dobson et al. found the incidence of allergic reactions for VAN 2.5 times greater than with other drugs (95% CI 0.979–5.70, *p* = 0.0374) [56]. Blumenthal et al. reported a HR of 1.66 for developing eosinophilia (95% CI 1.22–2.26) [48]. Kovacic et al. reported that 20% of VAN user experienced an infusion-related reaction, with half of these leading to change of therapy [73].

#### 2.5.4. Discontinuation and Readmission Due to Adverse Events

To assess the severity of VAN-induced adverse events, discontinuation or readmission due to adverse events are relevant outcomes.

Three studies reported on changes in VAN therapy or discontinuation due to AEs (Table 1) [60,61,89]. Discontinuation rates of 5.5–42.9% were reported.

Conflicting results regarding readmission risks are reported by the included studies. Three studies reported a significant risk of readmission with VAN use, risks ranged from 1.72 to 2.45 (expressed as aRR, OR) [25,45,95]. Four studies reported not a significant risk for readmission in VAN patients (OR 0.75, OR 1.16, IRR 0.88, aHR 1.5) [14,69,83,86]. Notably, Epperson et al. reported lower rates of readmission in only VAN patients who were monitored by a pharmacist-driven OPAT monitoring service (19.4% vs. 39.1%, *p* = 0.004) [58].

## 3. Discussion

This is the first systematic review reporting on the effectiveness and safety outcomes of vancomycin therapy in the outpatient setting. Our findings indicate that vancomycin can be effective in this context. Comparative studies between C-I and I-I of VAN demonstrated no significant differences in clinical cure rates. The safety profile of VAN in the outpatient setting is variable. Reported AE rates ranged widely from 5.7% to 85.7%. Nephrotoxicity, a known adverse event of vancomycin, was the most cited adverse event. Comparative studies suggest a higher risk during I-I therapy compared to C-I; however, these findings were not consistent. Two studies reported lower nephrotoxicity rates in AUC-based monitoring compared to trough monitoring. Reported line-related AE ranged from 1.1% to 5.7%. Discontinuation of VAN due to AEs occurred in 5.5% to 42.9% of cases, reflecting a potentially significant impact on treatment continuity. Readmission risks associated with VAN use were inconsistent across studies; while some reported elevated risks, others did not.

Across the included studies we observed a large variation and/or limited reporting on monitoring practices including TDM, OPAT delivery models, and OPAT teams. The observed wide ranges in clinical success and adverse event rates reflect true clinical diversity in the published literature rather than methodological flaws in our review process. Definitions of clinical success, adverse events, nephrotoxicity, and monitoring protocols differed widely, and follow-up durations varied considerably. This may have impacted treatment and adverse event outcomes, thereby hampering their interpretation and limiting the ability to draw firm conclusions about the expected clinical trajectory or safety profile of vancomycin during OPAT. Rather than reflecting true differences in efficacy or safety, much of the observed variation likely represents differences in study design, surveillance intensity, and outcome reporting practices. A wide variety of different OPAT delivery models were used in the included studies. These differences may influence treatment outcomes, as there may be a difference in monitoring and follow-up of patients. Especially the existence of a designated specialized OPAT team, as recommended by guidelines, varied among the studies or was often not described, further contributing to different treatment outcomes. Moreover, the reported incidences and risks of AE rates may be underrepresented, because in most studies, researchers focused on a limited scope of AEs. Nephrotoxicity, a known potential adverse effect of VAN, was frequently reported as an ADE associated with VAN use. However, baseline creatinine levels were often not available or not described in the studies, making the interpretation of developing renal injury related to VAN use difficult. Due to this variation in practices and limited reporting, we were only able to describe the results. By separating the studies performed in VAN patients from general OPAT studies, we have tried to limit this effect. The studies that were performed in VAN patients were designed and tailored for VAN treatment, thereby allowing for a meaningful comparison with inpatient VAN populations. To strengthen future research and improve comparability across studies, standardized outcome reporting is needed. At minimum, vancomycin OPAT studies should provide clear and consistent definitions for clinical success, nephrotoxicity, line-related complications, and readmission. Additionally, transparency regarding OPAT program structure, including monitoring frequency, team composition, follow-up processes, and applying standardized TDM protocols—including AUC-based dosing and continuous infusion of vancomycin—would allow more meaningful interpretation of safety and effectiveness outcomes, but also would increase vancomycin’s effectiveness and safety in OPAT settings.

A strength of this review is that we included both studies specifically on patients treated with VAN as well as studies on general OPAT outcomes reporting VAN outcomes as supporting evidence. Given our objective to conduct a broad review of the literature evaluating this, the search was not restricted to only include certain study designs, interventions, or durations. We did not apply strict inclusion and exclusion criteria leading to a large heterogeneity in study designs and interventions and heterogeneity in OPAT models, populations, indications, monitoring conditions, outcomes, etc. This approach allowed us to provide a good reflection of real-time practices. A future step would be to conduct a meta-analysis of the results with restrictive inclusion/exclusion criteria to compare VAN use with other antibacterial agents in the outpatient setting. Lastly, the predominance of retrospective cohort studies and lack of randomized controlled trials usually limits the strength of the evidence and contribute to potential bias. However, by assessing the quality of the included studies with the Newcastle–Ottawa scale, the quality of evidence can be assessed properly. The results of these assessments show that the quality of the included studies was considered fair to good. Nevertheless, randomized controlled trials evaluating vancomycin effectiveness and safety in OPAT settings are still needed.

This review demonstrates that vancomycin, while showing a high effectiveness rate in the outpatient setting, is associated with a risk for developing AEs. Several studies mention that monitoring by a dedicated OPAT team can reduce readmission rate in VAN patients [25,26,58,71]. Previous studies have demonstrated that patients discharged with medication subjected to TDM, have an increased risk for readmission during OPAT [24,25,26]. This emphasizes the need for establishing a specialized, multidisciplinary OPAT team, as recommended by the IDSA [2]. The team would assess the appropriateness of an OPAT prescription, monitor patients including laboratory follow-up with TDM, and subsequently intervene at an early stage during the OPAT trajectory. This review also shows the need for implementing an OPAT outcome registry for collecting data related to the OPAT service. The registry can monitor practice, standardize OPAT services, and improve quality of care [18,101]. In addition, without systematic tracking of OPAT use and outcomes, it is likely that prescribers underestimate the rate of AEs among patients receiving OPAT. This review highlights the importance of tailored vancomycin management in the outpatient setting to maximize effectiveness while minimizing adverse outcomes.

## 4. Materials and Methods

This review was conducted according to PRISMA (Preferred Reporting Items for Systematic reviews and Meta-Analyses) guidelines (Supplementary File S1) [27]. The International Prospective Register of Systematic Reviews (PROSPERO) registration number is CRD42023351365.

### 4.1. Search Strategy

A systematic literature search of the electronic databases of Embase, Medline ALL, Web of Science Core Collection and Cochrane Central Register of Controlled Trials was performed from inception until 20 March 2026. The search was limited to human studies and restricted to English-language publications. The full search strategies are reported in Supplementary File S2. After the search, duplicates were removed using a citation management software (Endnote VX9. Clarivate Analytics, Philadelphia, PA, USA). Reference lists of included studies and review articles were manually searched for additional publications.

### 4.2. Eligibility Criteria and Definitions

Both randomized controlled trials and non-randomized studies (both prospective and retrospective) published in peer-reviewed journals were included. Exclusion criteria were studies reporting only non-clinical outcomes and studies lacking independent vancomycin outcome data (i.e., studies not providing independent outcome data specifically attributable to vancomycin, these studies reported outcomes for the entire OPAT population as a whole, without separating results by antimicrobial agent. It was not possible to extract data that reflected vancomycin-specific safety or efficacy). Case-reports, data reported within guidelines, conference presentations, and letters without formal publication were excluded. Studies reporting the same data on the same cohort were used once by including only the most recently published article.

OPAT was defined as the administration of outpatient parenteral antimicrobial therapy without intervening hospitalization. Outpatient can be referring to a variety of settings, such as the home setting (hospital in the home, HITH), (skilled) nursing facility, infusion center, physician’s office, hospital-based ambulatory-care clinic, emergency department (ED), hemodialysis unit, long-term care facility and rehabilitation centre^18^. Every type of administration (e.g., self-administration (S-OPAT), administration by caregiver or (visiting) nurse) was allowed. In this review, only intravenously (IV) administered vancomycin was included.

Adverse events were categorized into line-related events and adverse drug events (ADEs). Line-related events included mechanical complications (dislodgement, occlusion/clotting, malfunction, leakage) or infectious complications (catheter-related bloodstream infections, site irritation/infection (phlebitis)). Adverse events reported by health care professionals as well as self-reported adverse events by the patient were included.

### 4.3. Study Selection

All studies were reviewed in duplicate and independently by two investigators (M.H. and R.C.). First, a screening was performed based on titles and abstract. Subsequently, the full texts of the selected articles were evaluated for final inclusion, the reasons for exclusion were recorded. All discrepancies across both steps were resolved through consensus.

### 4.4. Outcome Measures

The primary objectives were to assess the effectiveness and safety of VAN in OPAT services. The primary effectiveness outcome was rate of treatment success of the infection. The primary safety outcomes included rates and risks of: (i) nephrotoxicity, (ii) line-related adverse events, (iii) other adverse events, (iv) discontinuations due to adverse events, (v) readmissions due to adverse events.

### 4.5. Data Extraction

Data were extracted in duplicate and independently by two investigators (M.H. and R.C.) using a standardized form. Data items extracted included study (author, year), study design, population characteristics (sample size, sex, and age participants), OPAT therapy (antimicrobial drugs, continuous or intermittent VAN, VAN dosage, concomitant use of OPAT drugs), indication for OPAT, OPAT treatment duration, OPAT delivery model, monitoring (laboratory/TDM monitoring), VAN effectiveness outcomes, and safety outcomes. Conflicts in data extraction were resolved by recruiting a third author (H.O.) to attain consensus.

### 4.6. Quality Assessment

No randomized controlled trials (RCTs) were available for inclusion in this review. The Newcastle–Ottawa scale (NOS) for non-randomized studies was used to assess the quality of the included studies performed in VAN patients (Table 1) [102]. Studies performed in general OPAT population (Table 2) were not subjected to quality assessment, given that these studies were not primarily performed in VAN patients and these studies were only included in this review as supporting evidence. The NOS yields a maximum score of nine for questions regarding selection, comparability, and exposure. Studies were rated as having a high (<5), moderate (5–7), or low risk of bias (≥8). To be able to compare the quality of the included studies, a modified version of the NOS was used to assess the quality of the non-comparative studies. The scale was modified to remove the ‘comparability’ domain and ‘selection of the non-exposed cohort’ item [103]. The maximum score of the scale was reduced from 9 to 6. Conversion of these scores was modified to the following: ‘High’ (2 or 3 in selection domain AND 2 or 3 in outcome domain), ‘Moderate’ (1 in selection domain AND 2 or 3 in outcome domain), and ‘Low’ (0 in selection domain OR 0 or 1 in outcome domain) [55]. All risk of bias assessments were conducted independently in duplicate by two reviewers (M.H. and R.C.). Disagreements were resolved by recruiting a third author (H.O.) to attain consensus.

### 4.7. Data Synthesis and Analysis

Because of the heterogeneity in study methodology and outcome reporting and absence of RCTs, a quantitative analysis of data was deemed inappropriate. Hence, a qualitative synthesis of the data was consequently completed.

## 5. Conclusions

This systematic review suggests that vancomycin can be an effective agent to use in OPAT setting, although its use is associated with a risk of adverse events. Given the observational nature and heterogeneity of the included studies, our findings should be interpreted with caution. Safe outpatient use depends on improved robust monitoring TDM strategies, coordinated multidisciplinary oversight, and more consistent outcome reporting. Overall, the results highlight the potential value of a dedicated multidisciplinary OPAT team to support follow-up and individualized vancomycin management in the outpatient setting, while underscoring the need for higher-quality evidence.

## Figures and Tables

**Table 1:** Characteristics and main results of included studies performed in vancomycin patients.

Study (Author, Year), Study Design	Number of VAN Patients, VAN Dose	Age and Sex Patients	Concomitant Therapy	OPAT Indication	OPAT Duration	OPAT Delivery Model	Monitoring (Laboratory/TDM)	VAN Efficacy Outcomes	VAN Safety Outcomes	Other Outcomes
Benefield 2023, retrospective chart review ^1^ [28]	n = 374 I-I patients, n = 118 C-I patients Total median daily dose at discharge: 2000 mg (IQR 1500–3000) in I-I, 2500 mg (IQR 2000–3100) in C-I	I-I: mean 58 years (SD 14), 58% male C-I: mean 50 years (SD 16), 66% male	Any concomitant nephrotoxic medication: 26% in I-I, 21% in C-I No concomitant antimicrobial medication: 48% in I-I, 50% in C-I	PJI (28%), osteomyelitis (22%), spondylodiscitis (14%), other SSTI (9%), septic arthritis (8%), endocarditis/endovascular infection (7%), other device or hardware-associated infection (6%)	Median 31 days (IQR 17–42)	Home (65%), rest to SNF	Weekly lab + TDM (for one third not performed)	-	-n = 160 patients (33%) possible nephrotoxic event, of these n = 38 probable nephrotoxic event -93 ADE led to VAN discontinuation in 89 patients (AKI (41%), immunologic reactions (29%), neutropenia/leukopenia (10%), tinnitus/hearing loss (5%)) -No difference in proportion of patients experiencing nephrotoxicity between C-I and I-I (9.3% and 7.2% resp., aHR 0.72 (95% CI 0.35–1.50)) -Proportion of patients experiencing ADE similar between C-I and I-I (18.6% vs. 17.9% resp., aHR 0.93 (95% CI 0.56–1.53)) -No difference in ED encounter, mortality or readmission between C-I and I-I	-Median creatinine clearance at discharge: 108 mL/min vs. 141 mL/min for I-I and C-I resp. -Creatinine concentrations within 60 days: median 16.3 mg/dL in I-I, 19.0 mg/dL for C-I -Estimated AUC at discharge: 430 mg·h/L (IQR 350–520) in I-I, 390 mg·h/L (IQR 310–480) in C-I -Median VAN plasma concentration within 60 days of discharge: 16.3 mg/L vs. 19.0 mg/L for I-I and C-I
Chambers 2020, retrospective quality assurance project ^2^ [29]	n = 223 C-I patients Mean daily dose 2 g (IQR 1.4–2.5) in no nephrotoxicity_50%_ group, 1.4 g (IQR 1.2–1.8) in nephrotoxicity_50%_ group	No nephrotoxicity_50%_ group: median 64 years (IQR 53–72), n = 128/215 (60%) male Nephrotoxicity_50%_ group: median 74 years (IQR 49–85), n = 6/8 (75%) male	Not significantly different: use of aminoglycoside, piptazo, furosemide, other diuretic, ACE inhibitor/ARB, other antihypertensive medicines including CCBs, NSAID, ≥5 regular medicines	Orthopedic prosthetic device (36%), BJI (30%), endocarditis/endovascular infection (7%), abscess/collection (6%), SSTI (6%), bacteremia without a focus (5%), empyema/pneumonia (4%), other (6%)	Median 19 days (IQR 10–29) in no nephrotoxicity_50%_ group, 15.5 days (IQR 6–24) in nephrotoxicity_50%_ group (*p* = 0.4577)	Supervision by ID physicians, run by specialist nurses	TDM twice-weekly, if stable reduced to once-weekly Target: 20–25 mg/L	-	-No nephrotoxicity_50%_ group: n = 215, nephrotoxicity_50%_ group: n = 8 -Nephrotoxicity_30%_: 26 patients	-Weighted-average serum concentration positive predictors for nephrotoxicity -Baseline creatinine not statistically different between nephrotoxicity group and no nephrotoxicity group (*p* = 0.3341)
El Nekidy 2019, retrospective chart review ^3^ [30]	n = 70 HD outpatients Loading dose 20–25 mg/kg (max 2 g), maintenance dose 1 g per HD session. Off-protocol dosing allowed	Mean 63.4 years (SD 15.6), 54.3% male	Combination antibiotics use in clinical cure group (42.3%) versus clinical failure group (57.7%) (*p* = 0.028)	Bacteremia (n = 24), SSTI (n = 35), osteomyelitis/diabetic foot (n = 39), UTI/peritonitis (n = 6)	-	Outpatient HD units affiliated with a single-center of a community-based hospital	TDM performed, target pre-HD trough 15–20 mg/L	-Clinical cure: n = 46 (65.7%); clinical failure: n = 24 (34.3%). -Type of infection independent predictor of VAN success.	-	No significant differences in the loading dose, maintenance dose, or pre-HD levels between the cured and failed groups
Gillett 2024, interrupted time series study ^4^ [31]	n = 63 pharmacist-driven AUC cohort, n = 60 trough-based monitoring AUC cohort: initial VAN dose median 28 mg/kg (IQR 18.5–34.9) Trough cohort: initial VAN dose median 26.2 mg/kg (IQR 18.3–34.9)	AUC cohort: median 64 years (IQR 56–76), 50.8% female Trough cohort: median 64 years (IQR 51–71), 43.3% female	AUC vs. trough cohort: ACEi/ARB/AR (27% vs. 23.3%), aminoglycosides (0% vs. 1.7%), loop diuretics (20.6% vs. 18.3%), piptazo (0% vs. 1.7%)	Bacteremia (n = 33), BJI (n = 59), CNS (n = 2), endocarditis (n = 14), pulmonary (n = 7), SSTI (n = 10), UTI (n = 5), other (n = 20)	AUC cohort: total 1816 days, trough cohort: total 1498 days	-	Weekly TDM + lab (serum creatinine, serum urea nitrogen, liver function tests, complete blood cell count with differential) AUC target: 400–600 mg·h/L Trough target: 10–20 mg/L	-	-Nephrotoxicity significantly lower in the AUC cohort (6.3% vs. 23.3%; *p* = 0.01). -No difference in composite 90-day all-cause mortality or readmission (33.3% vs. 38.3%; *p* = 0.56). -Significantly less vancomycin discontinuation due to AEs in AUC cohort (4.8% vs. 18.3%, *p* = 0.02)	Total TDM samples: AUC cohort (n = 205), trough cohort (n = 267)
Grattan 2021, retrospective cohort study [32]	n = 301 VAN courses	Median 60 years (IQR 45–68), 39.2% female	Combination therapy in 49.5% of courses	Infected joint (30.2%), SSTI (14.6%), osteomyelitis (13.3%), bacteremia (11.3%), surgical site infection (9%), endocarditis (7.3%), meningitis/epidural abscess (5.3%), intra-abdominal infection (4.3%), device related infection (2.7%), pneumonia with *S. aureus* (0.7%), other (1.3%)	Median 28 days (IQR 14–42)	Nurse-led virtual vancomycin clinic, therapy at home	Twice weekly, if stable, weekly TDM + lab (complete blood count, creatinine)	285/301 (94.7%) completed treatment	-ADE-related discontinuation: 33 patients (11.0%, 95% CI 7.7–15.1%). 15/33 (45.5%) due to renal toxicity (5.0%, 95% CI 2.8–8.1%). -18/33 other AE (neutropenia (n = 5)) -Treatment failure related readmission: 9/301 (3.0%), 3/9 IV line-associated bacteremia	-Totals of 34.2% target level trough 10–15 mg/L, 41.5% target level trough 15–20 mg/L, 24.2% target level trough 10–20 mg/L -Baseline creatinine did not significantly change (*p* = 0.68) -Trough levels did significantly increase (*p* = 0.001)
Hamad 2022, retrospective study [33]	n = 1419 patients	Median 54 years, 53.8% male	Common concomitant OPAT drugs: cephalosporins (20.5%), carbapenems (11.1%), penicillin (5.5%)	SSTI (53.2%), SSI (36.5%), BJI (32.1%), septicemia (26.9%)	-	Home (85.5%), remainder at outpatient infusion center	Most patients 64.4% weekly TDM for 40–80% of their OPAT course	-	Readmission with AKI in 385 patients (2.7%)	-21.3% of patients no record of VAN TDM during >7 days treatment -TDM was not associated with lower risk of readmission with AKI -CKD and concomitant penicillin associated with readmission with AKI (aOR 22.63 [95%CI 1.96–3.52], aOR 1.73 [1.21–2.49])
Ingram 2008, retrospective cohort study ^5^ [34]	n = 102 C-I patients	Mean 48.2 years (SD 17.6), 73.5% male	Concomitant exposure to aminoglycosides, loop diuretics, ACEI/ARB	BJI (66.7%)	Mean 23.8 days (SD 16.9)	-	Weekly TDM + lab (creatinine) Target 20–25 mg/L	-	16/102 (15.7%) nephrotoxicity	-Mean baseline creatinine 78 umol/L (SD 32.5) -Mean steady-state concentration 15.5 mg/L (SD 10.8) -Nephrotoxicity associated with hypertension, concomitant exposure to aminoglycosides or loop diuretics, and VAN concentration of ≥28 mg/L -Neither the cumulative dose nor the duration of VAN therapy was found to be a risk factor
Ingram 2009, retrospective cohort study ^6^ [35]	n = 167 patients C-I (n = 112) vs. I-I (n = 55) C-I: 42.1 g I-I: 21.8 g *p* ≤ 0.001	C-I: mean 46.8 years, 62.5% male I-I: mean 57.3 years, 60% male Age *p* ≤ 0.001	No statistically significant differences in concurrent exposure to nephrotoxic agents	BJI (n = 45), SSTI (n = 10)	C-I: 21.9 days (SD 10.6), I-I: 20 days (SD 21.3), *p* = 0.611	-	Weekly TDM + lab (creatinine)	-	-11.6% nephrotoxicity in C-I group versus 23.6% in I-I group, *p* = 0.067 -C-I associated with later onset of nephrotoxicity (*p* = 0.005)	-C-I lower baseline creatinine (mean 71.4 mol/L vs. 83.8 mol/L; *p* = 0.003) and higher outpatient VAN dose (42.1 g vs. 21.8 g; *p* ≤ 0.001) -Matched based on propensity score:
Krueger 2022, retrospective case-control study [36]	n = 130 I-I patients Initial cumulative dose in cases: 2.1 g/24 h, controls: 2.3 g/24 h (*p* = 0.32)	Cases: mean 51.4 years (SD 16.7), 62.2% male Controls: mean 54.5 years (SD 16.4), 64.5% male	Any nephrotoxicity medication: 78.4% in cases, 49.5% in controls (*p* = 0.0036)	Blood stream infection (n = 12), IE (n = 15), BJI (n = 66), SSTI (n = 11), CNS (n = 11), intra-abdominal infection (n = 6), UTI (n = 4), other (n = 5)	Total duration of treatment: cases mean 58.1 days (SD 77.3), controls mean 35.6 days (SD 22.3)	-	Weekly TDM + lab (metabolic panel, complete blood count with differential)	-	-n = 37 cases (AKI patients), n = 93 controls -Non-AKI ADR: 16.2% in AKI group, 12.1% in control group (*p* = 0.53) -Early discontinuation: 29.7% in AKI group, 6.5% in control group (*p* ≤ 0.001) -Readmission: 64.9% in AKI group, 30.1% in control group (*p* ≤ 0.001)	-Maximum trough lever higher in AKI group (*p* < 0.001), -Patients in AKI group more underlying CRD (*p* = 0.02) -Creatinine similar at time of hospital discharge (0.89 mg/dL vs. 0.81 mg/dL; *p* = 0.23)
Nolan 2023, retrospective study [37]	n = 15 C-I patients Median initial dose 2500 mg, adjusted to 1750 mg	Median age 58 years, 87% male	Ceftriaxone (n = 1 with stage 1 AKI), cefepime (n = 1 without AKI), piptazo (n = 1 without AKI)	-	-		TDM Target 15–25 mg/L	-	-33% (5/15) AKI (n = 3 stage 1, n = 2 stage 2) -n = 1 discontinuation of antibiotics in stage 2 AKI	AKI cohort median higher AUC 0–24/MIC versus without AKI (756 mg·h/L versus 490 mg·h/L)
Norton 2014, retrospective chart review ^7^ [38]	n = 155 C-I episodes Median total dose 63 g (IQR 52–86) in nephrotoxicity group, 62 g (IQR 36–90) in no nephrotoxicity group (*p* = 0.51)	Nephrotoxicity: median 59 years (IQR 51–70), 65% male No nephrotoxicity: median 59 years (IQR 45–70), 70% male	ACEi/ARB use: 77% in nephrotoxicity group, 43% in no nephrotoxicity group (*p* = 0.0016) Aminoglycoside use: 8% in nephrotoxicity group, 4% in no nephrotoxicity group (*p* = 0.40)	-	Median 16 days (IQR 7–28) in nephrotoxicity group, 22 days (IQR 12–31) in no nephrotoxicity group	-	Weekly TDM + lab (renal function + full blood examination) Target: 20–30 mg/L	-	-Nephrotoxicity in 26/155 (17%) patient episodes -ACEi/ARB [OR 9.78 (95% CI 3.1–39.4), *p* ≤ 0.001] and maximum vancomycin Css [OR 1.11 (95% CI 1.05–1.19), *p* ≤ 0.001] independent predictors of nephrotoxicity -Total VAN dose and duration not associated with nephrotoxicity	-Median maximum concentration: 37 mg/L (IQR 32–45) and 30 mg/L (IQR 26–34) in nephrotoxicity group and no nephrotoxicity group resp. (*p* ≤ 0.001) -Maximum concentration > 32 mg/L: 77% in nephrotoxicity group, 34% in no nephrotoxicity group (*p* ≤ 0.001)
Pai 2006, case cohort study [39]	n = 14 cases (VAN-induced neutropenia), n = 100 controls Total grams: cases 41 ± 29 g, controls 57 ± 71 g (not statistically different)	Cases: mean 39 years (SD 13), 71% male Controls: mean 49 years (SD 15), 50% male Age *p* = 0.01	Combination therapy not associated with development of neutropenia (*p* ≥ 0.1).	Osteomyelitis (n = 78), endocarditis (n = 14)	Median 24 days (range 2–43) for cases, median 37 days (range 2–151) for controls *p* ≤ 0.05	Home	Weekly lab (complete blood cell count with differential, transaminases, serum chemistry)	-	-Suspected neutropenia (n = 20) -Neutropenia: n = 14 (12%) (ten cases moderate, four cases severe) -No hospitalization or death in VAN-induced neutropenia -ADE-related discontinuation: n = 30, n = 20 related to suspicion of neutropenia	-Trough levels not associated with development of neutropenia -Mean total grams VAN not statistically different between cases and controls. -n = nine cases of nephrotoxicity
Rees 2022, retrospective cohort study ^8^ [40]	n = 53 patients AUC-based goal trough dosing n = 65 patients traditional trough dosing	AUC trough group: mean 59.9 years (SD 12.6), 56.6% male Trough group: mean 58.5 years (SD 12.8), 61.5% male	Combination therapy: not significantly different between two groups	-	AUC trough group: mean 35.6 days (SD 8.7) Trough group: mean 37.5 days (SD 11.1)	Home	Weekly TDM + lab Target: individualized goal trough range coinciding with an AUC of 400–600 mcg∙h/mL.	Treatment failure: 15.1% in AUC trough group, 24.6% in trough group (*p* = 0.201)	-AKI incidence: 5.7% in AUC trough group, 23.1% in trough group (*p* = 0.01) -Hospital readmission (13.2% in AUC trough group vs. 16.9% in trough group, *p* = 0.617) -Total number of regimen changer per patient: mean 1.13 (SD 1.1) in AUC trough group, mean 1.64 (SD 1) in trough group (*p* = 0.006)	-
Shakeraneh 2020, propensity score-matched retrospective cohort study ^9^ [41]	n = 74 matched patients in C-I and I-I cohort I-I: median daily dose 2500 mg (IQR 2000–3000). C-I: median daily dose 2375 mg (IQR 1500–3000)	I-I: mean 53.9 years (SD 14.2), 55.4% male C-I: mean 53.5 years (SD 13.9), 55.4% male	Use of concurrent nephrotoxins: 55.4% in C-I, 43.2% in I-I (*p* = 0.139)	BJI (n = 98), bloodstream (n = 20), CNS (n = 12), SSTI (n = 8), pulmonary (2), other (n = 8).	Median 35 days (IQR 17–38) in I-I, median 26.5 days (IQR 14–36) in C-I	OPAT clinic (affiliated with hospital) run by ID physician	Weekly TDM + lab Target trough 15–20 for I-I, target C-I 20–25	Clinical failure: 10/74 (13.5%) C-I vs. 17/74 (23%) I-I (*p* = 0.147)	-I-I 3.22-fold increase in nephrotoxicity (18.9% vs. 6.8%; OR 3.22 (95% CI 1.10–9.46); *p* = 0.027)) -CKD more common in I-I group (*p* = 0.044) -C-I VAN associated with a slower onset to nephrotoxicity	-Baseline creatinine and creatinine clearance similar in I-I and C-I -CKD more common in I-I (17.6% vs. 6.8%, *p* = 0.044) -Median trough and AUC within target -Number of VAN concentrations similar between the groups
Shi 2023, retrospective cohort study [42]	n = 115 I-I patients Total daily VAN dose 2230 mg (SD 1026)	Mean 61.7 years (SD 16.1), 44% male	Monotherapy 67.8%, concomitant parenteral antibiotics 31.3%, no concomitant oral antibiotics 73.9%	Osteomyelitis (33%), PJI (26.15), endovascular device infection (7%), CNS (6.1%), CRBSI or primary bacteremia (5.2%), SSTI (4.3%), native joint septic arthritis (3.5%), endocarditis (3.5%), other infections (7%)	Mean 3.9 weeks (SD 2)	-	Weekly TDM + lab Target AUC: 400–600 mg·h/L.	-	-Readmission in 22 patients (19.1%) -With AUC-based vancomycin dosing, eight patients had AKI -Each of these patients had AUCs at goal, half with troughs 10–15 and half with troughs 15–20	-
Thijs 2022, prospective observational study ^10^ [16]	n = 35 C-I episodes	Median 61 years (range 11–75), 65.7% male	-	BJI (85.7%), (catheter-related) blood stream infection (8.6%), (endo)vascular infection (5.7%)	Median 18 days (range 4–63)	HITH	Biweekly TDM + lab (CRP and renal function) VAN target 20–25 mg/L	100% clinical cure rate	-ADE rate: n = 2 (5.7%) (neutropenic fever (1), eosinophilia and DRESS (1)) -ADE-related readmissions: n = 2 (5.7%) -Catheter-related AE: n = 2 (5.7%) -Readmission due to line-related AE: n = 2 (5.7%)	-An amount of 68.5% of VAN levels in range (16.7% subtherapeutic, 14.8% supratherapeutic) -Median concentration 22.5 mg/L (range 6.6–32.0)
Verrall 2012, retrospective cohort study ^11^ [43]	n = 188 C-I patients, n = 56 I-I patients Weight based dosing	C-I: mean 49.8 years (SD 17.7), 77.1% male I-I: mean 63.1 years (SD 15.9), 71.4% male Age: *p* ≤ 0.0001	Concurrent antibiotics: 6.4% in C-I, 10.7% in I-I (*p* = 0.275)	Osteomyelitis (n = 123), joint (n = 19), SSTI (n = 25), bloodstream (n = 19), endocarditis (n = 7), other (n = 51)	C-I: median 30 days (IQR 18–41) I-I: median 32.5 days (IQR 16–47)	-	Weekly TDM + lab Weekly review (clinical examination, lab tests (full blood count, CRP). VAN TDM weekly (C-I target 15–25 mg/L, I-I target trough 15–20 mg/L)	Clinical failure: 21.3% in C-I, 30.4% in I-I (RR 0.701, 95% CI 0.432–1.136, *p* = 0.159), after exclusion of patients with subtherapeutic levels: RR 0.752 (95% CI 0.386–1.465, *p* = 0.410)	No significant difference in rates of unplanned readmission, unplanned extension of therapy, or change in antibiotics	-
Vuagnat 2004, prospective study ^12^ [44]	n = 21 I-I patients, n = 23 C-I patients Daily mean dosing: I-I 31.9 mg/kg (SD 12.5), C-I: 33.9 mg/kg (SD 12.9)	Mean 56.7 years	Rifampicin: n = 9 in I-I, n = 5 in C-I Ciprofloxacin: n = 2 in I-I, n = 4 in C-I	Osteomyelitis	-	Home	Weekly TDM + lab (leucocyte count, serum creatinine, C-reactive protein) Target trough or plateau: 20–25 mg/L	I-I: 77.8% cured, C-I: 94.4% cured (*p* = 0.3)	ADR-related discontinuation: 42.9% in I-I vs. 8.7% in C-I (*p* = 0.03)	-Creatinine increased by mean 0.4 umol/L/day in I-I group (*p* = 0.02) -Mean trough (21.7 ± 9.3) lower than mean plateau concentration (26.0 ± 6.1 mg/L)

Abbreviations: 95% CI: 95% confidence interval, ACE(i): angiotensin-converting-enzyme inhibitor, ADE: adverse drug event, ADR: adverse drug reaction, AE: adverse event, aHR: adjusted hazard ratio, AKI: acute kidney injury, aOR: adjusted odds ratio, ARB: angiotensin receptor blocker, AUC: area-under-the-curve, BJI: bone and joint infection, CCBs: calcium channel blockers, C-I: continuous infusion, CKD: chronic kidney disease, CNS: central nervous system, CRD: chronic renal disease, CRP: c-reactive protein, Css: steady-state concentration, DRESS: drug reaction with eosinophilia and systemic symptoms, ED: emergency department, HD: hemodialysis, HITH: hospital in the home, ID: infectious diseases, IE: infectious endocarditis, I-I: intermittent infusion, IQR: interquartile range, MIC: minimal inhibitory concentration, NSAID: non-steroidal anti-inflammatory drug, OPAT: outpatient parenteral antimicrobial therapy, OR: odds ratio, piptazo: piperacillin/tazobactam, PJI: prosthetic joint infection, RR: risk ratio, SD: standard deviation, SNF: skilled nursing facility, SSTI: skin and soft tissue infection, TDM: therapeutic drug monitoring, UTI: urinary tract infection. ^1^ Possible nephrotoxicity was defined as an increase in serum creatinine ≥ 0.5 mg/dL between consecutive measurements, or a 50% increase from baseline. Probable nephrotoxicity was defined as possible nephrotoxicity plus discontinuation of vancomycin before the intended treatment duration with accompanying chart documentation of a suspected ADE by the following ID provider. ^2^ Vancomycin-induced nephrotoxicity was defined as a rise in serum creatinine of ≥50% or 44 µmol/L (nephrotoxicity_50%_) during treatment from at least two consecutive measurements. Baseline serum creatinine was defined as the value recorded at changeover from intermittent to continuous infusion. Nephrotoxicity was defined as the first time the definition threshold was attained and subsequent data for the patient were not included. ^3^ Clinical cure was defined as the complete resolution of the clinical signs of infection (i.e., fever, leukocytosis, local signs of infection, negative microbiological cultures) and the documentation of clinical cure with discontinuation of vancomycin. Treatment failure was documented if the patient: (a) experienced persistent bacteremia (i.e., repeated blood culture with same bacteria identification), (b) had a recurrent infection within 30 days of completing the previous vancomycin therapy, (c) died within 30 days of starting vancomycin, (d) required further interventions such as amputation and/or wound debridement, or (e) had documentation of clinical failure. ^4^ Nephrotoxicity was defined as a serum creatinine increase by ≥0.5 mg/dL or ≥50% during outpatient vancomycin therapy. ^5^ Nephrotoxicity was defined as more than 50% increase in serum creatinine compared with baseline ^6^ Nephrotoxicity was defined as >50% increase in serum creatinine compared with baseline, which resulted in a dose reduction ^7^ Nephrotoxicity was defined as a change in serum creatinine ≥ 50% from OPAT baseline. Creatinine clearance (CL_CR_) was calculated using the Cockcroft–Gault formula ^8^ Treatment failure was defined as 90-day all-cause mortality, recurrent culture growth of gram-positive bacteria related to the primary source of infection within 90 days of discharge, or readmission within 90 days from time of discharge related to the primary source of infection of the index case. ^9^ Clinical failure was defined as unplanned readmission, extension of therapy beyond originally planned, or antibiotic change due to therapy failure. Nephrotoxicity was defined as a serum creatinine (S_cr_) increase of greater than 0.5 mg/dL or greater than 50% increase from baseline for two consecutive measurements while receiving vancomycin during OPAT. ^10^ Patients were assessed as clinically cured at the end of therapy with vOPAT in the case of absence of fever or local signs of infection and if there were no unplanned hospital readmissions for the same clinical problem, as well as no registration of the same infection up to one month after completion of vOPAT. Clinical failure was determined as relapse of infection during or within one month after completion of vOPAT. Patients temporarily readmitted, whether unplanned or not, due to vOPAT- or non-vOPAT-related problems and who nonetheless finished their vOPAT episode were still assessed as either clinically cured or failed according to the abovementioned definition. ^11^ Clinical failure was defined as an unplanned re-admission, extension of therapy beyond that planned at admission to OPAT or the need to change antibiotics because of clinical, biochemical or radiological deterioration of the infection being treated. ^12^ Patients were considered cured if they remained asymptomatic 12 months after completion of therapy.

**Table 2:** Characteristics and main results of the included studies performed in general OPAT populations.

Study (Author, Year), Study Design	Total Number of Patients, Number of VAN Patients, VAN Dose	Age and Sex (All Patients)	Concomitant Therapy (All Patients)	OPAT Indication (All Patients)	OPAT Duration (All Patients)	OPAT Delivery Model (All Patients)	Monitoring (Laboratory/TDM)	VAN Efficacy Outcomes	VAN Safety Outcomes	Other Outcomes
Agnihotri 2023, retrospective quasi-experimental study [45]	n = 428 patients, n = 149 VAN patients	Median 52–57 years (IQR 43.5–67); n = 229 male	-	BJI (n = 174), CNS (n = 47), SSTI (n = 35), G/UTI: (n = 38), intra-abdominal infection (n = 36), endocarditis (n = 12), pneumonia (n = 4), other (n = 82)	Median 25–30 days (range 12–41)	Home (n = 234), SNF (n = 79), subacute rehabilitation facility (n = 112), infusion center (n = 1)	TDM + lab	-	VAN independently associated with unplanned OPAT-related hospital readmission, OR 2.448 (95% CI 1.203–4.984), *p* = 0.014	-
Barnes 2021, case-control study [46]	n = 116 patients with readmission, n = 116 controls, n = 101 VAN patients	Readmission group: mean 54.5 years (range 18–91), 59% male Controls: mean 58.2 years (range 18–100), 55% male	Number of medications at discharge: mean 15.9 (range 3–42) in readmission group, 14.7 (range 2–34) in control group	Bacteremia/endocarditis (n = 57) diabetic foot infection (n = 31), osteomyelitis (n = 100), etc.	Readmission group: mean 38 days (range 14–56) Controls: mean 36 days (range 7–58)	Home (n = 160), SNF (n = 59), infusion center (n = 13)	-	-	n = 51 (44%) VAN readmissions, n = 50 (43%) VAN controls. VAN use not associated with readmission	Creatinine at discharge not different (*p* = 0.3)
Barr 2012, retrospective cohort study [47]	n = 2766 OPAT episodes	-	-	-	-	S-OPAT and infusion center	-	-	VAN use associated with higher rates of OLE, but no statistical significance: OR 3.0 (95% CI 0.5435–16.56, *p* = 0.460).	-
Blumenthal 2015, prospective cohort study ^1^ [48]	n = 824 patients, n = 314 VAN patients (38%)	Median 60 years (IQR 48–71), 60% male	Monotherapy (n = 515/824, 63%)	Orthopedic infections (56%), bacteremia (20%), SSTI (15%), endocarditis (10%), etc.	Median 41 days (IQR 31–45)	Home (n = 470, 58%), SNF (n = 339, 42%)	Weekly lab	-	-n = 95 VAN patients with eosinophilia, developing eosinophilia: HR 1.66 [1.22, 2.26] *p* = 0.001). -Probable DRESS syndrome (n = 3) -VAN associated with renal injury with eosinophilia (HR: 2.53, *p* < 0.0001) and any injury (rash, renal injury or liver injury) with eosinophilia (HR: 1.70, *p* = 0.0002)	-
Bradley 2023, retrospective cohort study [49]	n = 200 patients, n = 78 VAN patients	Mean age 49 years, 60% male	-	BJI (52%), primary bacteremia (26%)	37.7 vs. 29.2 days	Home	Laboratory monitoring	-	-30-day readmission in 13 (32.5%) VAN patients, *p* = 0.371 -10 AKI episodes (38.5%), all VAN patients	-
Browning 2022, prospective cohort study [50]	n = 5201 courses, n = 602 VAN courses (11.6%)	Median 61 years (IQR 47–75), n = 1582 (38%) female	Episodes with >1 antimicrobial agent administered 435 (10.5%)	PJI (13.9%), CF (9.9%), osteomyelitis (9.4%) etc.	Median 20 days (IQR 12–29)	S-OPAT (approx. half of admissions) or H-OPAT	Weekly review by ID consultant + weekly lab (full blood count, urea electrolytes creatinine, liver enzymes, and CRP)	-	-VAN risk of major AKI compared to benzylpenicillin: HR: 7.68; 95% CI, 2.91–20.3), *p* ≤ 0.001 -Risk of any major AE compared to benzylpenicillin: HR: 2.70; 95% CI, 1.53–4.76), *p* = 0.001	-
Brzozowski 2020, retrospective cohort study [51]	≥65 yr: n = 204 patients, <65 yr: n = 253 patients ≥65 yr group: monotherapy VAN (n = 58, 28.4%), VAN combination (n = 39, 19.1%). <65 yr group: monotherapy VAN (n = 83, 32.8%), VAN combination (n = 46, 18.2%)	≥65 yr group: median 75 years (IQR 69–81), 62.8% male <65 yr group: median 54 years (IQR 45–59), 58.9% male	-	Osteomyelitis (n = 157), endovascular infection (n = 75), BJI (n = 65), CNS (n = 47), SSTI (n = 25), other (n = 137)	≥65 yr group: median 31.0 days (IQR 17.0–38.0) <65 yr group: median 33.0 days (IQR 22.0–38.0)	≥65 yr group: nursing home (n = 143, 70.1%), home (n = 50, 24.5%), other (n = 11, 4.4%) <65 yr group: nursing home (n = 104, 41.1%), home (n = 127, 50.2%), other (n = 22, 8.7%)	-	-	VAN predictive of development of AE: aOR = 1.9; 95% CI = 1.2–3.1	-
Buehrle 2017, retrospective cohort study ^2^ [52]	n = 67 IVDU users, n = 22 VAN patients (33%)	Median 34.5 years (range: 19–63 years), 53% male	-	Endocarditis (52%), epidural abscess (7%), bacteremia (4%), SSTI (4%), etc.	-	Nursing facility (n = 46, 69%), home (n = 20, 30%), drug rehabilitation facility (n = 1, 1%)	-	Treatment success: 9/22 (40.9%); treatment failure 13/22 (59.1%), *p* ≥ 0.99	ADR: 6/22 (27%) (neutropenia/leukopenia (5), AKI (1))	-
Chambers 2019, retrospective cohort study [53]	n = 385 patients with n = 407 courses in 2015/6 cohort Glycopeptides (n = 45, 10%)	Median 61 years (range 13–95), n = 141 (35%) female	No dual therapy in glycopeptides patients	Cellulitis/bursitis (7%), osteomyelitis (31%), infected prosthesis/device (15%), septic arthritis (12%), endocarditis (6%), abscess (n = 5%), bacteremia (6%), etc.	Median 20 days (range 2–157)	S-OPAT (n = 83, 20%)	Weekly follow-up by nursing services + blood tests. Weekly virtual review by OPAT team TDM VAN twice weekly Target 20–25 mg/L	-	Rash (3), AKI (1)	-
Cheong 2008, quality improvement audit [54]	n = 673 patients, n = 714 courses n = 52 C-I VAN courses (7.1%)	Mean 52.6 years (SD 19.2), 55% of courses in males	Combination therapy (n = 26 courses, 3.6%)	Cellulitis (44%), orthopedic infections (10%), wound infections (8.8%), etc.	Median 5 days (IQR 3–7)	Visiting nurse model	Daily assessment by nurse + once/twice weekly blood monitoring + weekly medical review	-	ADR in 5/52 (9.6%) courses, of these two serious ADR	-
Deng 2024, retrospective observational cohort study [55]	n = 73 patients preintervention group with n = 39 (53.4%) VAN patients, n = 355 patients post intervention group with n = 110 (31%) VAN patients	Preintervention: median 52 years (IQR 47–67) Post-intervention: median 57 years (IQR 43.5–60.5)	-	BJI (n = 174), CNS (n = 47), SSTI (n = 35), G/UTI (n = 38), intra-abdominal (n = 36), others (n = 98)	Preintervention: median 30 days (IQR 19–41) Post-intervention: median 25 days (IQR 12–38)	Ambulatory n = 235, non-ambulatory n = 193	-	-	The use of VAN during 12-month baseline period identified as an independent predictor of readmission based on the prespecified change-in-estimate criterion	-
Dobson 2004, prospective study [56]	n = 770 patients, n = 1000 OPAT courses, n = 156 VAN courses	Median 51 years (range 3 months–91 years), n = 506 male	n = 868 courses monotherapy	Osteomyelitis (n = 215), CF (n = 140), septic arthritis (n = 128) etc.	Median 19 days (range 1–167 days)	Home (69.7%), S-OPAT (30.3%)	-	-	-Allergic reactions in 5.8% of VAN courses -Incidence of allergic reactions for VAN 2.5 times greater than that with other drugs (95% CI 0.979–5.70, *p* = 0.0374)	90% of all courses C-I
Duggal 2009, retrospective chart review [57]	n = 74 patients n = 49 VAN courses (56%)	Median 64 years (range 25–88), 54% male	Combination therapy (n = 11 patients, 15%)	PJI	Median 35 days (range 2–80 days)	CoPAT	Lab monitoring weekly, varied according to used antimicrobial	-	-No antimicrobial AE related readmissions. -3 AE’s: Rash (1), nausea/vomiting (1), AKI (2)	-
Edwards 2025, retrospective cohort study [46]	n = 9088 treatment courses. n = 958 VAN courses.	Mean 63.2 years (SD 14.4), 59% male		Neutropenia patients: osteomye litis (37/161; 23.0%), PJI (35/161; 21.7%), bacteremia (34/161; 21.1%), SSTI (34/161; 21.1%)	39 days (IQR, 21–50 days)		Once-weekly CBC with differential monitoring, but may vary depending on clinical scenario		n = 15 neutropenic events, incidence per 100 courses: 4.1 (95% CI 2.5–5.6) OR for developing neutropenia: 1.10 (95% CI 0.75–1.61), *p* = 0.62	
Epperson 2023, retrospective cohort study [58]	n = 243 patients in OPAT monitoring group, n = 156 patients in control group, n = 116 VAN patients	Median 54 years (IQR 43–64), 53% male	79% monotherapy	BJI (23%), bacteremia (33%), SSTI (29%), etc.	Median 24 days (IQR 11–37)	Home	Laboratory monitoring	-	Lower rates of readmission for VAN patients in OPAT monitoring program (19.4% vs. 39.1%, *p* = 0.004)	-
Esposito 2007, retrospective cohort study [59]	n = 239 patients, VAN < 5%	Range 11–80 years, 62.3% male	Combination therapy in 66.9% of cases (43.9% with two antibiotics, 23% with three antibiotics)	Osteomyelitis (52.3%), septic arthritis (18.8%), PJI (18.8%), spondylodiskitis (10%)	Mean 71.2 days (SD 39.3)	Hospital (51.5%), care facility (1.8%), clinic (7.9%), doctor office (0.8%), home (30.1%), S-OPAT (7.9%)	-	-	-3 ADR (23.1%) in VAN users: Rash (n = 2, 15.4%), leukopenia (n = 1, 7.7%)	AEs more frequent in combination therapy regimens (74.1% vs. 25.9%)
Faden 2009, retrospective chart review [60]	n = 82 courses, n = 7 VAN courses	Median 9.3 years (range 5 months–20 years), n = 26 male	-	Osteomyelitis (n = 39), abscesses (n = 30, wound infections (n = 3), etc.	VAN courses: mean 4 weeks	-	Weekly lab (monitoring for bone marrow toxicity, renal/hepatic toxicity) + VAN TDM (trough target < 11 μg/mL)	-	-ADE rate: 85.7% (6/7) -ADE-related discontinuation rate: 42.9%	-
Felder 2016, retrospective cohort study [24]	n = 337 patients, n = 147 VAN patients (44%)	Mean 55 years (range 19–87), 43% female	-	Orthopedic infection (86%), neurosurgical infection (14%)	Median 38 days	Home (61%), SNF (35%), outpatient infusion center (4%)	-	-	-OPAT complications: aRR 1.7 (95% CI 1.3–2.1), *p* ≤ 0.01 -Adverse antibiotic reaction: aRR 2.1 (95% CI 1.5–3.0), *p* ≤ 0.01. -OPAT-related hospital readmission: aRR 2.2 (95% CI 1.3–3.8), *p* ≤ 0.01 -Vascular access failure: aRR 1.5 (95% CI 0.9–2.4), *p* = 0.10 -27/31 AKI patients on VAN, 12 of those readmitted	-
Fernandes 2018, retrospective observational study [61]	n = 540 cases, n = 118 VAN cases (22%)	Median 11.6 years (IQR 6.3–15.5), 45% female	Combination therapy (21%)	Non-device-associated musculoskeletal (39%), device/surgery-associated infections (21%), CNS (13%), lyme (8%), endocarditis (4%), other (15%)	Median 30 days (IQR 22.5–43)	-	1–2 scheduled ID clinic visit. Weekly lab (CBC, urea nitrogen, creatinine, hepatic transaminases, erythrocyte sedimentation rate, C-reactive protein) Weekly TDM VAN	-	-27/118 (23%) antimicrobial discontinuation -24/118 (20%) antimicrobial related complications (leukopenia (5), rash (8), fever (3)) -3/118 (3%) IV access-related complication -45/118 (38%) unplanned outpatient healthcare visits -25/118 (21%) readmissions	-
Flaten 2023, retrospective chart review ^3^ [62]	n = 137 treatment courses, n = 41 VAN treatment courses (29.9%)	Median age 65 years	Oral and IV combination regimens in 8%	PJI	Median 53 days (IQR 45–77 days)	-	-	No association of IV therapy with treatment failure	-	-
Frieler 2021, prospective cohort study [63]	n = 26 patients, n = 54 OPAT episodes, n = 11 VAN courses	Mean 66 years (SD 15), 70% female	-	PJI	Mean 66 days (SD 26)	S-OPAT	Weekly clinical examination + lab testing	-	-No unplanned readmission -1 AKI	-
Hale 2017, retrospective chart analysis [64]	n = 144 patients, 40.3% VAN patients	Mean 55.6 years (SD 14.8), 52.1% male	-	BJI (38.9%), SSTI (18.8%), bacteremia (13.9%), etc.	Median 27 days (IQR 19.25–36.0)	Home (81%), remainder rehabilitation facility	TDM VAN weekly	-	19 AKI cases, 13 of these on VAN	-
Hoffman-Terry 1999, retrospective chart review [65]	n = 269 patients, n = 112 VAN courses	Mean 47 years (range 0–86), male 57%	Combination therapy (45%)	BJI (59%), endovascular infections (16%), abscesses (9%)	Median 42 days (range 3–141)	S-OPAT	2/3 times per week monitoring + weekly lab (total leukocyte counts, absolute neutrophil counts, absolute eosinophil counts, platelet counts, serum creatinine)	-	-14/112 (13%) with leukopenia (ten monotherapy VAN) -6/112 (5%) with neutropenia (four monotherapy VAN) -9/112 (8%) eosinophilia in VAN containing regimens -3/112 (3%) thrombocytopenia in VAN containing regimens -Nephrotoxicity: 9/112 (8%) (eight combination with aminoglycoside or amphotericin B) -*C. difficile* colitis: 1 VAN user. -Rash: 3/112	-
Htin 2013, retrospective cohort study [66]	n = 68 patients, n = 7 VAN patients	Median 68 years (range 21–93), 87% male	Gentamicin for synergism (n = 18)	Infective endocarditis	Median 24 days (range 4–42)	HITH	Daily review by nursing staff, weekly review by ID team + weekly lab	-	No readmissions in VAN users	-
Huang 2018, retrospective cohort study [67]	n = 200 patients, n = 62 VAN patients (31%)	Median 60 years, 65.5% male	Total number of IV antimicrobials: median 1 (IQR 1–1)	Osteomyelitis/septic arthritis (35.5%), SSTI (23.5%), genital/UTI (17.5%), pneumonia (10%), etc.	Median planned duration OPAT: 18 days (IQR 7–34)	Home (60%), SNF/SAR (40%).	-	-	12/62 readmitted, with one due to ADR-related readmission (AKI under 1 g every 12 h)	-
Huminer 1999, retrospective study [68]	n = 37 patients, n = 7 VAN patients	Mean 64.3 years (SD 16.1), n = 21 male	-	Infective endocarditis	VAN mean 28 days (range 14–40)	Home (n = 34), S-OPAT (n = 24)	Laboratory monitoring		Local complications: occlusion (1), thrombophlebitis (1)	-
Ibaraki 2024, retrospective pre-post cohort study [69]	n = 361 patients, n = 127 VAN patients	Median 63 years (IQR 52–72), 62.1% male	-	Bacteremia (17.7%), osteomyelitis (17.5%)	Median 14 days (IQR 8–33)	Home, SNF, dialysis centers	-	-	Readmission OR 1.16 (95% CI 0.71–1.91), *p* = 0.55	-
Ismail 2025, retrospective cohort study [50]	n = 162 HD patients n = 81 VAN patients VAN dosing 3 times per week after HD	Median 59 years (IQR, 51–69 years), 41% female		Bloodstream-related infection (51%), BJI (22%)	Median 42 days (IQR, 28–42 days)	Home (n = 118, 73%), SNF/SAR (n = 39, 24%), long-term care facility (n = 4, 2.5%), unknown (n = 1, 0.5%)			n = 44 patients not readmitted, n = 37 patients readmitted (*p* = 0.271)	
Keller 2018, prospective cohort study [70]	n = 339 patients, n = 89 VAN patients (26.3%)	Median 55 years (IQR 41–63), 46.9% female	Combination therapy (19.5%)	Bacteremia (20.9%), abdominal (8.9%), osteomyelitis (30.1%), septic arthritis (7.4%)	Mean 64.5 days, median 29 days (IQR 15–44)	S-OPAT	Weekly catheter dressing changes + lab	-	Catheter complications/1000 OPAT days: VAN use aIRR 2.32, 95% CI: 1.20–4.46	-
Keller 2018, prospective cohort study [71]	n = 339 patients, n = 89 VAN patients (26.3%)	Median 55 years (IQR 41–63), 46.9% female	Combination therapy (19.5%)	Uncomplicated bacteremia (20.9%), endocarditis or endovascular infection (7.1%), cellulitis (5.6%), osteomyelitis (30.1%), etc.	Median 29 days (IQR 15–44)	S-OPAT	-	-	-ADE: n = 22 (24.7%) -Clinically significant ADE: n = 19 (21.3%) (nine changed medication, five stopped all medication, five readmitted due to ADE) -ADEs: rash (1), elevated levels (n = 2), ototoxicity (n = 1), nausea (n = 2), *C. difficile* (n = 1), nephrotoxicity (n = 11), cytopenias (n = 6), drug fevers (n = 2), edema (n = 1) -VAN independent predictor for significant ADEs/1000 OPAT days (aIRR: 2.19, 95% CI: 1.78–5.72)	-
Kieran 2009, prospective study [72]	n = 56 patients, n = 21 VAN patients	Median 50 years (range 16–88), 57% male	Monotherapy (02%)	Musculoskeletal infection (50%), osteomyelitis (42%), septic arthritis (8%)	Median 16 days (range 2–84)	H-OPAT (n = 12 courses), S-OPAT (n = 48 courses)	Once–twice weekly for clinical assessment + TDM VAN	-	Switch to teicoplanin due to drug rash (2)	Difficulty achieving therapeutic levels in 5/21 (23%)
Kovacik 2023, retrospective case series [73]	n = 115 first-dose infusions, n = 20 VAN first-dose infusions	Median 60 years (51–67), 40% female	-	BJI (68%), SSTI (24%)	-	Infusion center	-	-	n = 4 (20%) of VAN infusions leading to an infusion-related reaction, 54% of these led to a change of therapy	-
Lai 2013, retrospective chart review [74]	n = 393 OPAT courses, n = 147 VAN courses (37.4%)	Mean 62 years, n = 328 male	12.7% combination IV therapy	Osteomyelitis foot (28.8%), bacteremia (19.3%), osteomyelitis non foot (10,9%), PJI (9.2%), UTI (5.6%), endocarditis (5.3%), etc.	Mean 21.1 days (IQR 9–30)	S-OPAT or administration by healthcare professional	Weekly calls + nurse visits for PICC dressing changes + VAN TDM (target trough 15–20 mg/L)	-	-ADEs: 16/147 (11%) -AKI (8/16, 5 discontinuations, all reversible), leukopenia (4/16)	8.1% of patients ESRD and/or HD
Lam 2023, retrospective cohort study [75]	n = 2513 courses, n = 541 VAN courses	Median 64 years (IQR 51–74), 37.1% female	Two IV antibiotics concurrently in 24.9% cases, concomitant oral antibiotics in 13.4%	Osteoarticular infection (58.4%), bacteremia (18.8%), abscess (11.9%	Median 6 weeks (IQR 6–6)	-	-	-	- A total of 21 cases (3.9%) of neutropenia -Combined incidence per 100 courses: 5.6 (95% CI 3.8–7.9)	Median neutrophil count at neutropenia diagnosis higher in VAN patients versus other antibiotic classes, *p* = 0.05
Le 2010, retrospective cohort study [76]	n = 66 patients without complications, n = 32 patients with complications n = eight VAN patients	Without complication: mean 8.2 years (SD 5.1), n = 38/66 male With complication: mean 6.3 years (SD 5.1), n = 20/32 male	Combination therapy in group without complications (n = 23), with complications (n = 12)	Osteomyelitis (n = 50), joint infection (n = 10), pneumonia (n = 25), CF (n = 12), SSTI (n = 1)	Without complications: mean 21 days (SD 10.6) With complications: mean 27 days (SD 15.2)	Home	-	-	5 VAN patients without complications, 3 VAN patients with complications, *p* = 0.519	-
Li 2018, prospective study [77]	n = 3435 patients, n = 118 VAN patients	Median 51 years (range 4–99), 58.7% male	-	SSTI (61.3%), BJI (15.3%), UTI (4.5%), bacteremia (8.9%), etc.	Median 4 days (range 1–78)	HITH	Weekly outpatient medical review	-	Three ADR-related readmissions	-
Lin 2005, retrospective chart review [78]	n = 177 patients, n = 11 VAN regimens	-	-	Chronic rhinosinusitis	6–8 weeks	-	-	-	Monotherapy VAN: Septicemia (1), flushing (1)	-
Manzella 1985, retrospective study [79]	n = 52 courses, n = 7 VAN courses	Mean 45 years	Combination therapy in several cases	Osteomyelitis (n = 28), endocarditis (n = 6), Hickman catheter infections (n = 4), miscellaneous (n = 7)	Mean 20.5 days (range 3–99)	S-OPAT	Two times/week examination by ID physician + IV-line check. Lab when deemed necessary	-	0 AEs	-
Means 2016, retrospective cohort study [80]	n = 216 patients, n = 45 VAN patients (20.8%)	Median 55.5 years (IQR 44–64), 54.2% male	-	BJI (31.9%), CNS (9.7%), endocarditis (14%), etc.	Median 14 days (IQR 8–35)	Home (57.4%), SNF (34.7%), subacute rehabilitation facility (6%), infusion center (1.9%)	-	-	n = 15 (34.9%) readmitted versus n = 30 (17.3%) not readmitted, *p* = 0.011 in univariate analysis. Multivariate analysis: not significant.	-
Ng 2021, retrospective chart review [81]	n = 602 patients, 16% VAN	-	69.1% monotherapy, 25.7% dual therapy, 5.2% receiving ≥3 agents.	Osteomyelitis (34.4%), bacteremia (24.8%), SSTI (16.4%), PJI (10.6%), etc.	-	78.0% home, 11.3% SNF, 6% long-term acute-care hospital, 4.2% daily infusion center	-	-	-12.4% transaminase elevation -19% electrolyte abnormalities -17.4% creatinine abnormalities -22.3% leukopenia	-
Olson 2014, retrospective cohort study [82]	n = 335 children, n = 62 VAN patients (18.5%)	Median 7.4 years (IQR 2.3–13.3), 60% male	Monotherapy (34.0%), dual therapy (27.2%), triple therapy (13.4%), >4 antibiotics (25.4%)	Septic arthritis (17.6%), acute osteomyelitis (15.2%), chronic osteomyelitis (7.8%)	Total IV treatment duration median 24 days (IQR 8–44)	-	Weekly lab (CBC, creatinine, liver function tests), 90.5% received laboratory monitoring	-	-AE: n = 6/62 (9.7%), -Serious AE: n = 3/62 (4.8%)	-
Palms 2020, retrospective cohort study [83]	n = 755 patients, n = 236 VAN patients (31%)	Median 58 years (IQR 45–67), 57.4% male	-	BJI (44.7%), bloodstream infection (23.2%), cardiovascular (12%), etc.	Planned duration: 30 days (IQR 18–37)	Home (84.2%), rehabilitation facility (15.8%)	Weekly lab	-	n = 36 readmitted (263%) versus n = 200 not readmitted (n = 32.4%), OR 0.75 (95% CI 0.49–1.13), *p* = 0.17	Only antimicrobial class associated with readmission was anti-fungal
Pulcini 2008, retrospective study [84]	n = 129 patients, n = 92 VAN patients (71%)	Mean 54 years (SD 18), 71% male	47% patients received parenteral-only antimicrobial therapy	Chronic bone infection	Total parenteral treatment duration mean 133 days (SD 100)	HITH	Individualized clinical monitoring + lab (CBC, serum liver markers, creatinine levels)	-	-36% AE rate, OR 1.17 (95% CI 0.52–3.62, *p* = 0.71) -Antibiotic-related complication: allergy (n = 4), nephrotoxicity (n = 3), neutropenia (n = 3, grade 1/2/4), vestibular toxicity (n = 1)	-
Schechter 2023, retrospective cohort study [85]	n = 106 courses, n = 41 courses (39%)	Median 51 years (IQR 42–56), 76%	76% courses included ≥2 antibiotics	Diabetic foot osteomyelitis	Median total duration 42 days (IQR 31–43)	Home-based (94%)	Weekly lab monitoring	-	AKI in 13/41 (37%)	-
Schmidt 2017, retrospective cohort study [86]	n = 2228 patients, n = 443 VAN users	19–30 years: 7.3% 31–40 y: 8.4%, 45–50 y: 16.1%, 51–60 y: 23.1%, 67–70 y: 23.9%, >70 y: 21.1%, 57.7% male	-	Cellulitis/wound infection/abscess (20.5%), postoperative infection (19.3%), bacteremia (20.9%), osteomyelitis (18.3%), etc.	<14 days (33.1%), 14–42 days (56.9%), >42 days (9.9%)	Home (61.4%), SNF (23.1%), infusion center (7.6%), dialysis center (3.0%), rehabilitation facility (4.9%)	-	-	-IRR for any unplanned hospitalization within 90 days: 0.88 (95% CI 0.60–1.27, *p* = 0.49) for glycopeptides (VAN) use -16/443 patients developed AKI (with three on combination therapy).	-
Seetoh 2013, prospective cohort study [14]	n = 2229 first episodes (total)	Median 56 years (IQR 43–67), 64% male	-	Among others: osteomyelitis (15%), endocarditis	Median 16 days (IQR 8–27)	Hospital OPAT (76%), S-OPAT (17%), homecare OPAT (7%)	-	-	No risk of deterioration (unplanned readmission): aHR = 1.5, 95% CI 0.9–2.5, *p* = 0.141	-
Shrestha 2016, retrospective cohort study [87]	n = 1461 patients, n = 496 VAN patients	Mean 55 years (SD 16), n = 842 male	Monotherapy (n = 1263), dual therapy (n = 189), therapy with 3 agents (n = 9)	Abdominal infection (n = 219), BJI (n = 363), bacteremia (n = 174), CNS (n = 108), chest/respiratory infection (n = 104), endocarditis and cardiac device infection (n = 235), SSTI (n = 195), etc.	Median 20 days (IQR 10–34, range 1–176)	Home (62%)	-	-	Catheter occlusion: 38/1000 OPAT days, thrombosis 4/1000 OPAT days	-VAN: 12,460 OPAT days -The number of intravenous antibiotics per OPAT course was not associated with the occurrence of vascular access complications
Skogen 2024, retrospective study [88]	n = 170 patients, n = 7 VAN patients	Median 64 years (range 19–93)	-	Endocarditis (n = 18), BJI (n = 53), postoperative infections (n = 46), other infections (n = 43)	Median 13 days	Infusion center	Laboratory tests at least twice a week at the hospital along with catheter control and observation of general condition	-	One allergic reaction during treatment (no hospital readmission or death)	-
Tice 2001, retrospective study [89]	n = 4000 patients (US OPAT Outcomes Registry)	-	-	Osteomyelitis	-	-	-	-	ADR-related discontinuation: 5.5% of VAN courses	-
Tice 2003, retrospective chart review [90]	n = 454 patients VAN: 1 g BID or OD (for impaired renal clearance)	-	-	Osteomyelitis	VAN: mean 34 days	Ambulatory infusion center	-	*S. aureus* infections treated by VAN: RR 2.5 (95% CI 1.1–5.7; *p* = 0.03) compared with PRP	-	-
Townsend 2018, retrospective study [91]	n = 107 patients, n = 49 VAN courses	Mean 54.1 years, 57% male	-	Musculoskeletal (52.2%), endovascular (37.4%), CNS (8.4%)	Mean 31.2 days (range 6–130)	HHC (62%), SNF (38%)	ID clinics visit 1–3 weeks after discharge, lab weekly	-	ADEs in n = 10/49 courses (20%)	-
Townsley 2021, retrospective cohort study [92]	n = 181 courses, n = 30 VAN courses (16.6%)	Median 6.7 years (IQR 1.6–13.2), 45.3% female	Combination therapy (15.5%)	BJI (24.9%), SSTI (16.6%), CNS (24.9%), bloodstream infection (39.2%), etc.	Median 12 days (IQR 8–27)	Home	Clinical follow-up at ID clinic + lab	-	-Any AE (n = 12, 40%), no AE (n = 18, 60%), *p* = 0.870 -Risk for the development of any AE for VAN versus other antibiotic: OR 0.27, 95% CI 0.08–0.88, *p* = 0.031	-
Voumard 2018, prospective observational study ^4^ [93]	n = 150 patients, n = 32 VAN patients	Median 59 years (range 6–93), 72% male	-	Osteo-articular infections (53%), endovascular (12%), urinary (11%), etc.	Median 13 days (range two to one hundred and four)	S-OPAT (82%), administration by home health nurse (13%), administration in OPAT unit (4%), mixed (1%)	TDM at least once weekly of all agents	Treatment failure: n = 2/32 (6%)	-AE: n = 5 (16%) (febrile agranulocytosis (1), PICC thrombosis (1), neutropenia grade 1 (2), acute renal failure grade 1 (1))	Mean VAN concentration: 17.2 mg/L (SD 5.3)
Wang 2023, retrospective observational cohort study [94]	664 patients	57% male	-	Bloodstream (30.7%), BJI (31.5%), CNS (5.3%), etc.	-	-	-	-	VAN risk factor for adverse events: OR 1.107 (95% CI 1.069–1.144)	-
Wee 2020, prospective cohort study [95]	n = 1213 total referrals	-	-	Primary bacteremia, prosthetic infection, osteomyelitis, intra-abdominal abscess, pyelonephritis	-	S-OPAT or hospital ambulatory setting	-	-	-Complications: with using VAN versus not using VAN: aRR 1.97 (95% CI 1.26–3.06), *p* = 0.003 -Early OPAT termination requiring readmission with using VAN versus not using VAN: aRR 1.72 (95% CI 1.03–2.88), *p* = 0.037	-
Wynn 2005, retrospective analysis [96]	n = 1515 patients, n = 515 VAN patients VAN: mean dose 1641 mg/d (range 500–4000) in 383 treatment courses	Mean 52.3 years (range 1–92), n = 817 male	Monotherapy	Wound infections (n = 331), SSTI (n = 273), acute osteomyelitis (n = 165), septic arthritis/bursitis (n = 153), bacteremia (n = 100), etc.	-	-	-	-	-43/515 (8.3%) AEs -Anaphylaxis (3), rash (20), leukopenia (5), nausea/vomiting (3), renal toxicity (4), diarrhea (3), rash/fever (1), other (4) -AE related discontinuation: compared to ceftriaxone (*p* = 0.001), cefazolin (*p* = 0.021), nafcillin (*p* = 0.610), oxacillin (*p* = 0.020), clindamycin (*p* = 0.531)	-
Yan 2016, retrospective chart review [97]	n = 104 patients, n = 14 VAN patients (13%)	Median 63 years (IQR 43–74), 63% male	Some patients received combination therapy (oral and parenteral concurrently)	Surgical site infection (33%), osteoarticular infection (28%), bacteremia (21%), etc.	-	Home	-	-	Return to ED or readmission (n = 6) versus no return to ED or readmission (n = 8), *p* = 0.79	-

Abbreviations: 95% CI: 95% confidence interval, ADE: adverse drug event, ADR: adverse drug reaction, AE: adverse event, aHR: adjusted hazard ratio, aIRR: adjusted incidence rate ratio, AKI: acute kidney injury, aOR: adjusted odds ratio, aRR: adjusted risk rate, BID: bis in die, BJI: bone and joint infection, CBC: complete blood count, CF: cystic fibrosis, C-I: continuous infusion, CNS: central nervous system, CoPAT: community-based outpatient parenteral antimicrobial therapy, CRP: c-reactive protein, DRESS: drug reaction with eosinophilia and systemic symptoms, ED: emergency department, ESRD: end stage renal disease, G/UTI: genital/urinary tract infection, HD: hemodialysis, HHC: home healthcare companies, HITH: hospital in the home, H-OPAT: home OPAT, HR: hazard ratio, ID: infectious diseases, IQR: interquartile range, IV: intravenous, IRR: infection related readmission, IVDU: intravenous drug users, OD: once daily, OLE: other line events, OPAT: outpatient parenteral antimicrobial therapy, OR: odds ratio, PICC: peripherally inserted central catheter, PJI: prosthetic joint infection, PRP: penicillinase-resistant penicillin, RR: risk ratio, SAR: subacute rehabilitation, SD: standard deviation, SNF: skilled nursing facility, S-OPAT: self-administration outpatient parenteral antimicrobial therapy, SSTI: skin and soft tissue infection, TDM: therapeutic drug monitoring, UTI: urinary tract infection, VAN: vancomycin. ^1^ Renal injury was defined as a creatinine increase of at least 0.5 mg/dL or 50% above baseline creatinine. ^2^ Treatment failure was defined as worsening or ongoing infection requiring hospital readmission within 30 days, worsening or ongoing infection resulting in prolonged antibiotic therapy, antibiotic noncompliance, noncompliance with follow-up clinic appointments, or death during treatment course. ^3^ Treatment failure was defined as readmission to the OPAT program for infection of the same joint, additional surgery outside of the original treatment plan, extension of IV antibiotic treatment beyond 8 weeks, persistence of symptoms, readmission to hospital for reasons related to the infection, and loss to follow-up before completion of treatment. ^4^ The patients were considered cured in the case of absence of fever, no local signs of infection at the end of the treatment assessed by an infectious disease specialist and no unplanned readmission to our hospital for the same cause within 3 months after the end of treatment. Unplanned readmissions during OPAT, relapses of infection during or after the end of OPAT, or deaths during or within the 3 months after the end of OPAT were treatment failures. Expected readmissions, such as, e.g., for an elective change of a prosthesis, were not considered treatment failures.

## Data Availability

No new data were created or analyzed in this study.

## Supplementary Materials

The following supporting information can be downloaded at: https://www.mdpi.com/article/10.3390/antibiotics15060630/s1, Supplementary File S1: PRISMA_2020_checklist; Supplementary File S2: Search strategy and search results; Supplementary File S3: Newcastle Ottowa Scale.
