# Supplementary material for: Clinical Outcomes and Safety Profile of Vancomycin in Outpatient Parenteral Antimicrobial Therapy Services: A Systematic Review
Source: Antibiotics (Basel). 2026 Jun 22;15(6):630. doi: 10.3390/antibiotics15060630 (PMC13296339; doi:10.3390/antibiotics15060630)
Supplement: Supplementary file 1 [file antibiotics-15-00630-s001.zip › Supplementary file 2 Search strategy and search results.pdf]

Supplementary file 2

Search strategy and results

Embase 432

('vancomycin'/de OR (vancomycin* OR vancocin*):ab,ti,kw) AND ('home infusion therapy'/de OR 'outpatient parenteral antimicrobial therapy'/de OR 'outpatient parenteral antibiotic therapy'/de OR (('outpatient'/de OR 'outpatient care'/de OR 'outpatient department'/de OR 'home care'/de) AND ('infusion therapy'/de OR 'parenteral drug administration'/de OR 'vancomycin'/'parenteral drug administration' OR 'intravascular drug administration'/de OR 'intravenous drug administration'/de)) OR (((home* OR outpatient* OR out-patient* OR domestic* OR domiciliar*) NEAR/3 (infusion* OR parenteral* OR intravascular* OR intravenous* OR IV) NEAR/3 (therap* OR service* OR treat* OR program*)) OR OPAT* OR c-OPAT* OR cOPAT*):ab,ti,kw) NOT ((animal/exp OR animal*:de OR nonhuman/de) NOT ('human'/exp)) AND [ENGLISH]/lim

Medline ALL Ovid 80

(Vancomycin/ OR (vancomycin* OR vancocin*).ab,ti,kw.) AND (Home Infusion Therapy/ OR ((Outpatients/ OR Ambulatory Care/ OR Home Care Services/ OR Home Care Services, Hospital-Based/) OR Drug Administration Routes/ AND (Home Infusion Therapy/ OR Infusions, Parenteral/ OR Injections, Intravenous/)) OR (((home* OR outpatient* OR out-patient* OR domestic* OR domiciliar*) ADJ3 (infusion* OR parenteral* OR intravascular* OR intravenous* OR IV) ADJ3 (therap* OR service* OR treat* OR program*)) OR OPAT* OR c-OPAT* OR cOPAT*).ab,ti,kw.) NOT ((exp animal/ OR animal* OR nonhuman/) NOT (exp human/)) AND english.lg.

Web of Science Core Collection*

TS=(((vancomycin* OR vancocin*)) AND (((home* OR outpatient* OR out-patient* OR domestic* OR domiciliar*) NEAR/2 (infusion* OR parenteral* OR intravascular* OR intravenous* OR IV) NEAR/2 (therap* OR service* OR treat* OR program*)) OR OPAT* OR c-OPAT* OR cOPAT*))

Cochrane Central Register of Controlled Trials

(((vancomycin* OR vancocin*)) AND (((home* OR outpatient* OR out NEXT patient* OR domestic* OR domiciliar*) NEAR/2 (infusion* OR parenteral* OR intravascular* OR intravenous* OR IV) NEAR/2 (therap* OR service* OR treat* OR program*)) OR OPAT* OR c NEXT OPAT* OR cOPAT*))

Table S1 Search results

Database searched	via	Years of coverage	Records	Records after duplicates removed
Medline ALL	Ovid	1946 - Present	117	117
Embase	Embase.com	1971 - Present	727	626
Web of Science Core Collection*	Web of Knowledge	1975 - Present	153	32
Cochrane Central Register of Controlled Trials	Wiley	1992 - Present	10	4
**Total**			**1007**	**779**

*Science Citation Index Expanded (1975-present) ; Social Sciences Citation Index (1975-present) ; Arts & Humanities Citation Index (1975-present) ; Conference Proceedings Citation Index- Science (1990-present) ; Conference Proceedings Citation Index- Social Science & Humanities (1990-present) ; Emerging Sources Citation Index (2015-present)
