# Supplementary material for: Clinical Outcomes and Safety Profile of Vancomycin in Outpatient Parenteral Antimicrobial Therapy Services: A Systematic Review
Source: Antibiotics (Basel). 2026 Jun 22;15(6):630. doi: 10.3390/antibiotics15060630 (PMC13296339; doi:10.3390/antibiotics15060630)
Supplement: Supplementary file 1 [file antibiotics-15-00630-s001.zip › Supplementary file 3 Newcastle Ottowa Scale.pdf]

Table S2 Newcastle-Ottawa quality assessment scale of included vancomycin cohort studies

Study/Year	Selection				Comparability	Outcome			Total score
	Representa tiveness	Selection of the non-exposed cohort	Ascertain ment	Endpoint does not present at start	Comparability (Confounding)	Assessment of outcome	Follow-up duration	Adequacy follow-up	
**Benefield 2023 [28]**	*	*	*			*	*	*	6
**Gillett 2024 [31]**	*	*	*			*	*	*	6
**Ingram 2009 [35]**	*	*	*		**	*	*		7
**Pai 2006 [39]**	*	*	*	*	*	*	*	*	8
**Rees 2022 [40]**	*	*	*		*	*	*	*	7
**Shakeraneh 2020 [41]**	*	*	*		**	*	*	*	8
**Verrall 2012 [43]**	*	*	*		*	*	*	*	7
**Vuagnat 2004 [44]**	*	*	*			*	*	*	6

Table S3 Newcastle-Ottawa quality assessment scale of included vancomycin cohort studies with modified Newcastle-Ottowa scale

Study/Year	Selection				Comparability	Outcome			Total score
	Representa tiveness	Selection of the non-exposed cohort	Ascertain ment	Endpoint does not present at start	Comparability (Confounding)	Assessment of outcome	Follow-up duration	Adequacy follow-up	

**Chambers 2020 [29]**	*		*			*	*		4
**El Nekidy 2019 [30]**			*			*	*		3
**Grattan 2021 [32]**	*		*			*	*	*	5
**Hamad 2022 [33]**	*		*	*		*	*	*	6
**Ingram 2008 [34]**	*		*			*	*		4
**Nolan 2023 [37]**	*		*			*	*	*	5
**Norton 2014 [38]**	*		*			*	*	*	5
**Shi 2023 [42]**	*		*	*		*	*	*	6
**Thijs 2022 [16]**	*		*			*	*	*	5

Table S4 Newcastle-Ottawa quality assessment scale of included vancomycin case-control studies with modified Newcastle-Ottowa scale

Study/Year	Selection				Comparability	Outcome			Total score
	Case definition	Representativeness the cases	Selection controls	Definition controls	Comparability (Confounding)	Ascertainment of exposure	Method ascertainment	Non-response rate	
**Krueger 2022 [36]**	*	*	*	*	**	*	*	*	9
